# United States State-Level Variation in the Use of Neuraxial Analgesia During Labor for Pregnant Women

**DOI:** 10.1001/jamanetworkopen.2018.6567

**Published:** 2018-12-28

**Authors:** Alexander J. Butwick, Jason Bentley, Cynthia A. Wong, Jonathan M. Snowden, Eric Sun, Nan Guo

**Affiliations:** 1 Department of Anesthesiology, Perioperative and Pain Medicine, Stanford University School of Medicine, Stanford, California; 2 Quantitative Sciences Unit, Stanford University School of Medicine, Stanford, California; 3 Department of Anesthesia, Roy J. and Lucille A. Carver College of Medicine, University of Iowa, Iowa City; 4 School of Public Health, Oregon Health & Science University–Portland State University, Portland

## Abstract

**Importance:**

Neuraxial labor analgesia is recognized as the most effective method of providing pain relief during labor. Little is known about variation in the rates of neuraxial analgesia across US states. Identifying the presence and extent of variation may provide insights into practice variation and may indicate where access to neuraxial analgesia is inadequate.

**Objective:**

To test the hypothesis that variation exists in neuraxial labor analgesia use among US states.

**Design, Setting, and Participants:**

Retrospective, population-based, cross-sectional analysis using US birth certificate data. Participants were 2 625 950 women who underwent labor in 2015.

**Main Outcomes and Measures:**

State-specific prevalence of neuraxial analgesia per 100 women who underwent labor and variability in neuraxial analgesia use among states, assessed using multilevel multivariable regression modeling with the median odds ratio and the intraclass correlation coefficient to evaluate variation by state.

**Results:**

In the study population of 2 625 950 women, 0.1% (n = 2010) were younger than 15 years, 7.0% (n = 183 546) were between the ages of 15 and 19 years, 23.6% (n = 620 118) were between the ages of 20 and 24 years, 29.6% (n = 777 957) were between the ages of 25 and 29 years, 26.0% (n = 683 656) were between the ages of 30 and 34 years, 11.4% (n = 298 237) were between the ages of 35 and 39 years, 2.2% (n = 57 130) were between the ages of 40 and 44 years, and 0.1% (n = 3296) were between the ages of 45 and 54 years. More than 90% were privately insured or insured with Medicaid. Neuraxial analgesia was used by 73.1% (n = 1 920 368) of women. After adjustment for antepartum, obstetric, and intrapartum factors, Maine had the lowest neuraxial analgesia prevalence (36.6%; 95% CI, 33.2%-40.1%) and Nevada the highest (80.1%; 95% CI, 78.3%-81.7%). The adjusted median odds ratio was 1.5 (95% CI, 1.4-1.6), and the intraclass correlation coefficient was 5.4% (95% CI, 4.0%-7.9%).

**Conclusions and Relevance:**

Results of this study suggest that a small portion of the overall variation in neuraxial analgesia use is explained by US states. Unmeasured patient-level and hospital-level factors likely account for a large portion of the variation between states. Efforts should be made to understand what the main reasons are for this variation and whether the variation influences maternal or perinatal outcomes.

## Key Points

**Question:**

Does the prevalence of neuraxial labor analgesia vary across US states?

**Findings:**

In this population-based, cross-sectional analysis of 2 625 950 pregnant women who underwent labor, Maine had the lowest adjusted neuraxial analgesia prevalence (36.6%) and Nevada the highest (80.1%). The odds of receiving neuraxial analgesia were 1.5-fold higher if the same patient received neuraxial analgesia in a high-use vs a low-use state; and 5.4% of the overall variation in neuraxial analgesia prevalence is explained by US state.

**Meaning:**

Results of this study suggest that wide variation exists in neuraxial analgesia use across US states, with a small portion of the overall variation explained by US states.

## Teaser

This population-based, cross-sectional analysis using US birth certificate data examines variation in prevalence of neuraxial labor analgesia among US states.

## Introduction

Pain relief for pregnant women in labor is commonly administered in the form of epidural, spinal, or combined spinal-epidural blockade (collectively referred to as neuraxial analgesia).^1^ Women receiving these techniques have lower pain scores and higher satisfaction scores and are less likely to require additional pain relief compared with women receiving systemic opioid analgesia.^2^ Guidelines for levels of maternal care and for obstetric analgesia and anesthesia published by the American College of Obstetricians and Gynecologists (ACOG) state that anesthesia services should be available to provide labor analgesia in all hospitals that offer maternal care.^3,4^ Although geographical variability has been shown for obstetric procedures, such as abortion availability,^5^ labor induction,^6^ cesarean delivery,^7^ and perinatal outcomes,^8,9^ there is insufficient research to determine whether geographical variation exists across US states in neuraxial analgesia use. Therefore, documenting and understanding geographical variability in the use of neuraxial analgesia are essential undertakings to improve the quality of obstetric anesthesia care.

Multilevel modeling is an approach that is well suited to characterizing the variability in the use of neuraxial analgesia, accounting for both patient-level and state-level factors, and to assessing the contribution of these factors to state-level variability in neuraxial analgesia use. We conducted a population-based analysis of women receiving neuraxial labor analgesia in the United States in 2015. We hypothesized that, after adjustment for patient case mix and state-level measures for the anesthesia workforce, the use of neuraxial analgesia would vary across US states.

## Methods

### Study Population and Primary Outcome

In this retrospective, population-based, cross-sectional analysis, we used US birth certificate data from 2015. These data are publicly available and deidentified; therefore, our analysis was deemed exempt from Stanford University Institutional Review Board approval. This study followed the Strengthening the Reporting of Observational Studies in Epidemiology (STROBE) reporting guideline. We examined data sourced from birth certificates for 100% of US births from 49 states and the District of Columbia (96.5% of all births in the United States in 2015).^10^ These states use the 2003 revised US Standard Certificate of Live Birth format. We did not examine 2015 birth data for Connecticut because the state did not use the 2003 revised US Standard Certificate of Live Birth format. The 2003 revised format contains detailed and consistent demographic, medical, and obstetric data, including information on methods of intrapartum analgesia.^11^ Data are entered into the birth certificate by each birth facility according to clinical records and maternal surveys. Details of variables included in the revised birth certificate have been published by the Centers for Disease Control and Prevention.^12^ Each birth certificate contains a check box to indicate whether or not the patient received “epidural or spinal anesthesia during labor.” No specific details are included in the birth certificate for the type of neuraxial block (epidural, spinal, or combined spinal-epidural blockade) or other analgesic modalities, such as systemic opioids or inhaled nitrous oxide.

**Figure 1.**
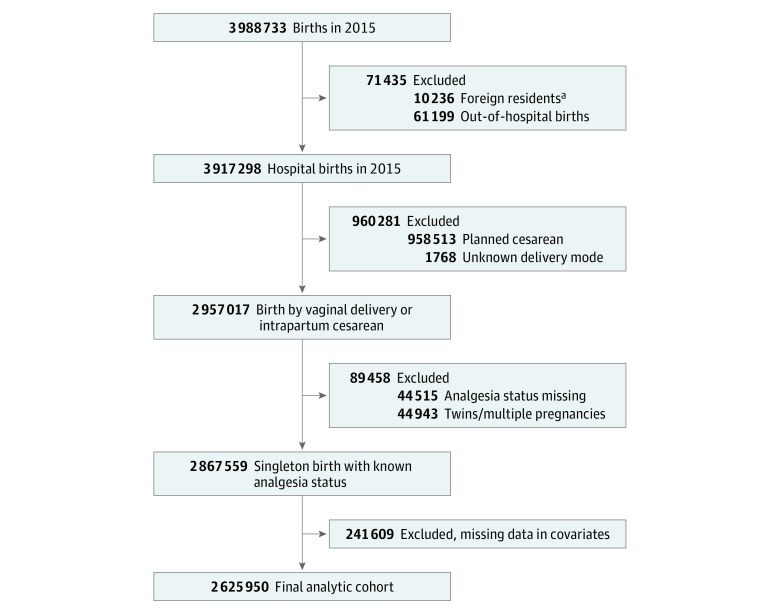
Study Flowchart ^a^Foreign residents are classified by the Centers for Disease Control and Prevention as women residing in a state that is not 1 of the 50 US states or District of Columbia.

Our study population included women who experienced labor before vaginal delivery or intrapartum cesarean delivery. We excluded women who had a non-US primary residence, an out-of-hospital delivery, multiple pregnancy, cesarean delivery without prior labor, and missing data for mode of delivery or neuraxial labor analgesia use. The final study population comprised 2 625 950 women (Figure 1). We used a complete case analysis (the highest percentage of missing values among all covariates was 2.5% for body mass index [BMI]). The frequencies of missingness for each variable by state are listed in the eTable in the Supplement. The primary outcomes were state-specific prevalence of neuraxial analgesia per 100 women who underwent labor and variability in neuraxial analgesia use among states, assessed using multilevel multivariable regression modeling with the median odds ratio (MOR) and the intraclass correlation coefficient (ICC) to evaluate variation by state.

### Patient-Level Factors

The following patient-level factors were used to build a multivariable model: maternal age, race/ethnicity, insurance type, highest level of education, marital status, BMI at delivery (classified using the World Health Organization BMI categories^13^), prepregnancy diabetes, prepregnancy hypertension, prior live birth, previous cesarean delivery, trimester when prenatal care was initiated, gestational diabetes, gestational hypertension or preeclampsia, eclampsia, gestational age at delivery, labor augmentation, labor induction, birth attendant, and fetal presentation. Details of the patient-level factors are listed in Table 1.

**Table 1.** Patient Characteristics and Measures of Association Between Individual and Hospital Characteristics and Neuraxial Labor Analgesia Use

Variable	No. (%)			Model 3 Multilevel Logistic Regression Adjusted OR (95% CI)^a^
	Total Study Population	Women Using Neuraxial Labor Analgesia	Women Not Using Neuraxial Labor Analgesia	
**Patient Characteristics**				
Maternal age, y				
<15	2010 (0.1)	1541 (0.1)	469 (0.1)	1.71 (1.53-1.90)
15-19	183 546 (7.0)	140 945 (7.3)	42 601 (6.0)	1.17 (1.16-1.19)
20-24	620 118 (23.6)	463 646 (24.2)	156 472 (22.2)	1.10 (1.09-1.10)
25-29	777 957 (29.6)	569 055 (29.6)	208 902 (29.6)	1 [Reference]
30-34	683 656 (26.0)	493 142 (25.7)	190 514 (27.0)	0.96 (0.95-0.97)
35-39	298 237 (11.4)	210 061 (10.9)	88 176 (12.5)	0.93 (0.92-0.94)
40-44	57 130 (2.2)	39 654 (2.1)	17 476 (2.5)	0.90 (0.88-0.92)
45-54	3296 (0.1)	2324 (0.1)	972 (0.1)	0.90 (0.83-0.97)
Race/ethnicity				
Non-Hispanic white	1 409 509 (53.7)	1 081 556 (56.3)	327 953 (46.5)	1 [Reference]
Hispanic	613 435 (23.4)	401 407 (20.9)	212 028 (30.1)	0.75 (0.75-0.76)
Non-Hispanic Asian	160 897 (6.1)	117 559 (6.1)	43 338 (6.1)	0.99 (0.98-1.01)
Non-Hispanic black	358 007 (13.6)	261 844 (13.7)	96 163 (13.6)	0.86 (0.85-0.87)
Non-Hispanic other	84 102 (3.2)	58 002 (3.0)	26 100 (3.7)	0.81 (0.80-0.83)
Insurance type				
Private	1 276 859 (48.6)	980 724 (51.1)	296 135 (42.0)	1 [Reference]
Medicaid	1 150 808 (43.8)	809 383 (42.1)	341 425 (48.4)	0.81 (0.81-0.82)
Other	112 405 (4.3)	81 355 (4.2)	31 050 (4.4)	0.94 (0.92-0.95)
Self-pay	85 878 (3.3)	48 906 (2.6)	36 972 (5.2)	0.54 (0.53-0.55)
Highest level of education				
Grade ≤8	87 181 (3.3)	44 232 (2.3)	42 949 (6.1)	0.57 (0.56-0.58)
Grade 9-12 with no diploma	298 463 (11.4)	202 227 (10.5)	96 236 (13.6)	0.87 (0.87-0.88)
High school graduate or GED completed	668 690 (25.5)	480 676 (25.0)	188 014 (26.6)	1 [Reference]
Associate degree	213 058 (8.1)	160 546 (8.4)	52 512 (7.4)	1.11 (1.10-1.13)
Some college credit but not a degree	563 712 (21.5)	426 204 (22.2)	137 508 (19.5)	1.14 (1.13-1.15)
Bachelor’s degree	506 740 (19.3)	386 178 (20.1)	120 562 (17.1)	1.14 (1.13-1.15)
Master’s degree	224 024 (8.5)	171 049 (8.9)	52 975 (7.6)	1.15 (1.13-1.16)
Doctorate or professional degree	64 082 (2.4)	49 256 (2.6)	14 826 (2.1)	1.21 (1.19-1.24)
Marital status				
Married	1 532 444 (58.4)	1 123 508 (58.5)	408 936 (58.0)	1 [Reference]
Unmarried	1 093 506 (41.6)	796 860 (41.5)	296 646 (42.0)	1.12 (1.11-1.13)
Body mass index at delivery^b^				
Normal or underweight	322 187 (12.3)	223 910 (11.6)	98 277 (13.9)	1 [Reference]
Overweight	943 032 (35.9)	678 751 (35.3)	264 281 (37.5)	1.10 (1.09-1.11)
Obesity class I	751 399 (28.6)	552 265 (28.8)	199 134 (28.2)	1.19 (1.17-1.20)
Obesity class II	370 724 (14.1)	279 889 (14.6)	90 835 (12.9)	1.26 (1.25-1.28)
Obesity class III	238 608 (9.1)	185 553 (9.7)	53 055 (7.5)	1.33 (1.31-1.34)
Prepregnancy diabetes	15 431 (0.6)	12 097 (0.6)	3334 (0.5)	1.13 (1.08-1.18)
Prepregnancy hypertension	35 540 (1.4)	28 575 (1.5)	6965 (1.0)	1.14 (1.10-1.17)
No prior live birth	1 142 006 (43.5)	920 899 (48.0)	221 107 (31.3)	1.76 (1.75-1.77)
Previous cesarean delivery	86 051 (3.3)	66 047 (3.4)	20 004 (2.8)	1.87 (1.84-1.90)
Trimester when prenatal care was initiated				
First	2 022 474 (77.0)	1 502 462 (78.2)	520 012 (73.7)	1 [Reference]
Second	449 955 (17.1)	315 881 (16.5)	134 074 (19.0)	0.87 (0.87-0.88)
Third	117 100 (4.5)	81 490 (4.2)	35 610 (5.0)	0.87 (0.86-0.89)
No prenatal care	36 421 (1.4)	20 535 (1.1)	15 886 (2.3)	0.61 (0.59-0.62)
Gestational diabetes	136 329 (5.2)	102 916 (5.4)	33 413 (4.7)	1.04 (1.02-1.05)
Gestational hypertension or preeclampsia	138 358 (5.3)	114 564 (6.0)	23 794 (3.4)	1.19 (1.17-1.20)
Eclampsia	4790 (0.2)	3941 (0.2)	849 (0.1)	1.15 (1.06-1.24)
Gestational age at delivery, wk				
37-42	2 459 720 (93.7)	1 807 692 (94.1)	652 028 (92.4)	1 [Reference]
24-36	162 016 (6.1)	111 332 (5.8)	50 684 (7.2)	0.82 (0.81-0.83)
<24	4214 (0.2)	1344 (0.1)	2870 (0.4)	0.17 (0.16-0.18)
Labor augmentation	710 290 (27.0)	586 970 (30.6)	123 320 (17.5)	2.31 (2.30-2.33)
Labor induction	819 874 (31.2)	685 271 (35.7)	134 603 (19.1)	2.31 (2.29-2.33)
Birth attendant				
Physician	2 320 775 (88.4)	1 739 608 (90.6)	581 167 (82.4)	1 [Reference]
Midwife	289 862 (11.0)	171 635 (8.9)	118 227 (16.7)	0.54 (0.54-0.55)
Other	15 313 (0.6)	9125 (0.5)	6188 (0.9)	0.61 (0.59-0.63)
Fetal presentation				
Cephalic	2 581 813 (98.3)	1 887 973 (98.3)	693 840 (98.3)	1 [Reference]
Breech	14 907 (0.6)	10 768 (0.6)	4139 (0.6)	1.19 (1.15-1.24)
Other	29 230 (1.1)	21 627 (1.1)	7603 (1.1)	1.07 (1.04-1.10)
**Anesthesia Workforce Measures**				
No. of anesthesiologists per 1000 births^c^				
6.12-12.02	250 948 (9.6)	173 428 (9.0)	77 520 (11.0)	1 [Reference]
12.09-13.91	640 216 (24.4)	482 377 (25.1)	157 839 (22.4)	1.50 (1.06-2.14)
14.14-15.85	300 363 (11.4)	239 475 (12.5)	60 888 (8.6)	1.59 (1.12-2.25)
15.87-18.62	847 667 (32.3)	585 548 (30.5)	262 119 (37.1)	1.20 (0.83-1.73)
19.64-35.23	586 756 (22.3)	439 540 (22.9)	147 216 (20.9)	1.05 (0.74-1.48)
No. of CRNAs per 1000 births^c^				
4.58-8.49	727 396 (27.7)	513 135 (26.7)	214 261 (30.4)	1 [Reference]
8.53-12.41	371 557 (14.1)	263 353 (13.7)	108 204 (15.3)	0.80 (0.56-1.14)
12.62-21.19	684 418 (26.1)	519 062 (27.0)	165 356 (23.4)	1.06 (0.74-1.51)
23.32-29.87	488 127 (18.6)	354 759 (18.5)	133 368 (18.9)	0.87 (0.61-1.25)
30.19-38.31	354 452 (13.5)	270 059 (14.1)	84 393 (12.0)	0.96 (0.66-1.36)

Abbreviations: CRNAs, certified registered nurse anesthetists; GED, general equivalency diploma; OR, odds ratio.

^a^ Adjusted for maternal age, race/ethnicity, insurance type, highest level of education, marital status, body mass index at delivery, prepregnancy diabetes, prepregnancy hypertension, prior live birth, previous cesarean delivery, trimester when prenatal care was initiated, gestational diabetes, gestational hypertension or preeclampsia, eclampsia, gestational age at delivery, labor augmentation, labor induction, birth attendant, number of anesthesiologists per 1000 births, and number of CRNAs per 1000 births.

^b^ Body mass index (calculated as weight in kilograms divided by height in meters squared) categories are as follows: normal or underweight (<24.9), overweight (25-29.9), obesity class I (30-34.9), obesity class II (35-39.9), and obesity class III (≥40).

^c^ Ranges are quintiles.

### Anesthesia Workforce Measures

The anesthesia workforce may influence the use of neuraxial labor analgesia; therefore, measures of the anesthesia workforce were incorporated into our model. Based on an approach described by Chang et al,^14^ we quantified the anesthesia workforce as the number of clinicians available for a given population in each state. We developed 2 measures of the anesthesia provider workforce, one for physician anesthesiologists and another for certified registered nurse anesthetists (CRNAs). Data for the total number of clinicians in each state were sourced from the 2015 US Health Resources and Services Administration Area Health Resource File.^15^ Using these data, we calculated ratios of clinicians to births for each state as the number of physician anesthesiologists and CRNAs per 1000 births, respectively.

### Statistical Analysis

Multilevel logistic regression was performed with the GLIMMIX procedure in SAS (version 9.4; SAS Institute Inc) using maximum likelihood estimation based on the Laplace approximation.^16^ Three regression models were fit sequentially. First, we estimated an unconditional or “null” model that included state as a random effect. In the null model (model 1), the random effect may be interpreted as each state’s deviation from the mean state-level neuraxial analgesia rate. In model 2, patient-level factors were added to determine whether the state-level variation remained after accounting for patient-level factors. In model 3, state-level anesthesiologists and CRNAs workforce measures were added to model 2 to determine how much of the state-level variation can be explained by anesthesia workforce measures. Because of a nonlinear association between each state-level workforce measure and our outcome, and for greater ease in interpretation, we categorized each workforce measure using quintiles.

For each model, we summarized the between-state variation using the random-effects variance, the ICC, and the MOR.^17,18,19,20^ The ICC represents the proportion of the total variance in neuraxial analgesia use attributable to US states after accounting for differences in patients (case mix). The MOR is interpreted as the median value of the odds ratio between a state with a higher likelihood of neuraxial analgesia use and the state with the lower likelihood when randomly picking 2 states from the sample. In our study, the MOR indicates the extent to which the individual probability of neuraxial analgesia is determined by state and is directly comparable to the odds ratios for patient-level factors. Details of the calculations of state-level adjusted prevalence, the ICC, and the MOR are presented as eMethods in the Supplement. We calculated 95% CIs for the MOR and the ICC.

We used the estimated random effects from model 1 and model 2 (before and after adjustment for patient-level factors) to describe the variation in neuraxial analgesia prevalence across states. These data are presented as heat maps and caterpillar plots.

Indications for neuraxial analgesia are not available in the birth certificate, and some patients who underwent intrapartum cesarean delivery may have received de novo neuraxial blockade for surgical anesthesia. Therefore, to assess the robustness of our findings, we repeated our primary analysis among women who underwent vaginal delivery and women who underwent intrapartum cesarean delivery separately. Statistical analysis was performed with SAS (version 9.4; SAS Institute Inc), and figures were generated in R (version 3.3; R Foundation for Statistical Computing).

## Results

We identified 3 988 733 births in 2015. We excluded 1 031 716 deliveries because of residency outside of the United States, out-of-hospital births, cesarean deliveries without labor, and unknown delivery modes. We also excluded 44 515 deliveries with missing data for neuraxial use and 44 943 twins or higher multiple pregnancies. Finally, we excluded 241 609 women who had missing data in any covariates. A flow diagram of women who met our exclusion criteria and our final analytic sample is shown in Figure 1. Our final analytic sample comprised 2 625 950 deliveries. In the study population of 2 625 950 women, 0.1% (n = 2010) were younger than 15 years, 7.0% (n = 183 546) were between the ages of 15 and 19 years, 23.6% (n = 620 118) were between the ages of 20 and 24 years, 29.6% (n = 777 957) were between the ages of 25 and 29 years, 26.0% (n = 683 656) were between the ages of 30 and 34 years, 11.4% (n = 298 237) were between the ages of 35 and 39 years, 2.2% (n = 57 130) were between the ages of 40 and 44 years, and 0.1% (n = 3296) were between the ages of 45 and 54 years. More than 90% were privately insured or insured with Medicaid. Among our final analytic sample, 1 920 368 (73.1%) received neuraxial labor analgesia. Table 1 lists descriptive characteristics of the final analytic sample.

Table 1 lists the characteristics of women who did and did not receive neuraxial analgesia, with the adjusted odds ratios from the multilevel model that included patient-level factors and anesthesia workforce measures (model 3). Intrapartum factors most strongly associated with neuraxial analgesia were labor augmentation and labor induction. Women with a previous cesarean delivery were more likely to receive neuraxial analgesia compared with women with no history of cesarean delivery. Compared with women with prior live births, women with no prior live birth were more likely to receive neuraxial analgesia. Sociodemographic factors inversely associated with neuraxial analgesia were older maternal age, nonwhite race and Hispanic ethnicity, no private insurance or no insurance, twelfth grade or less as the highest level of education, and late or no prenatal care. Compared with physician birth attendants, the odds of receiving neuraxial analgesia were reduced for midwives and other birth attendants. We observed no clear “dose-response” association between the number of anesthesiologists per 1000 births or the number of CRNAs per 1000 births with neuraxial analgesia.

### Variation in Statewide Prevalence of Neuraxial Analgesia Use

**Figure 2.**
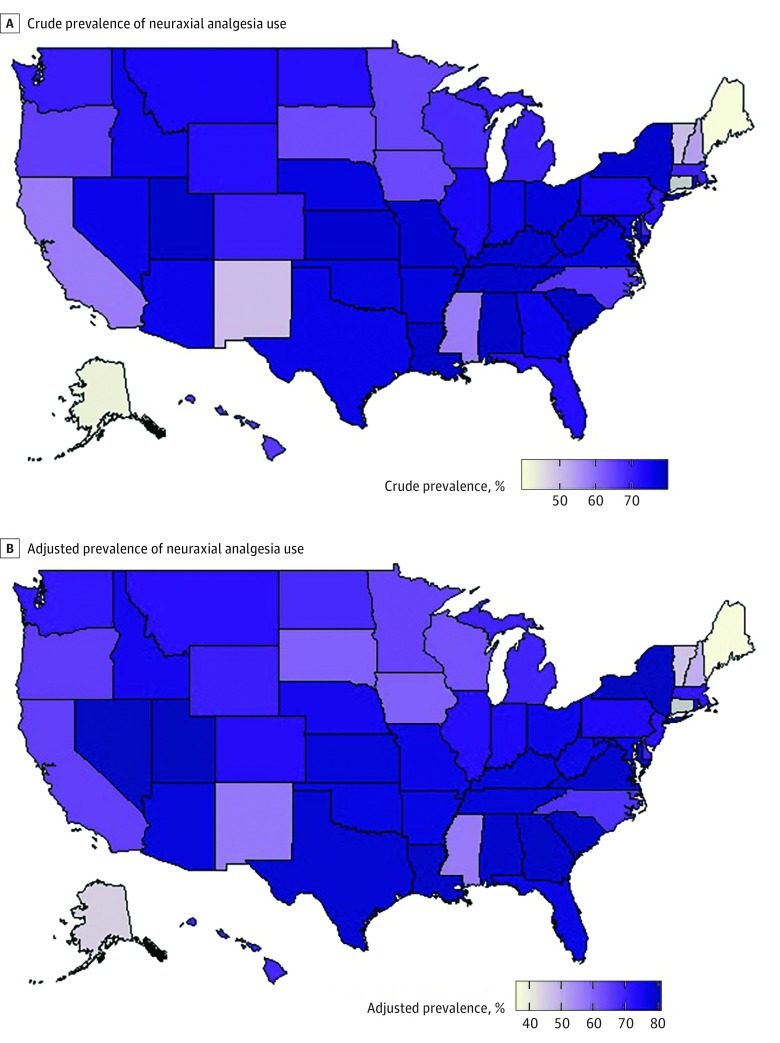
State-Specific and Adjusted State-Specific Prevalence of Neuraxial Analgesia Use in 2015 A, Heat map of crude state-specific prevalence of neuraxial analgesia use across US states in 2015. B, Heat map of adjusted state-specific prevalence of neuraxial analgesia use across US states in 2015. Connecticut did not provide data.

Figure 2 shows heat maps for the prevalence of neuraxial analgesia by state before and after adjustment for patient-level factors, respectively. Caterpillar plots are shown in eFigure 1 and eFigure 2 in the Supplement. After adjustment for antepartum, obstetric, and intrapartum factors, Maine had the lowest adjusted neuraxial analgesia prevalence (36.6%; 95% CI, 33.2%-40.1%) and Nevada the highest (80.1%; 95% CI, 78.3%-81.7%). The adjusted MOR was 1.5 (95% CI, 1.4-1.6), and the ICC was 5.4% (95% CI, 4.0%-7.9%).

Table 2 lists the MORs and ICCs of the 3 models. The MOR in model 1 was 1.5 (95% CI, 1.4-1.6), indicating that women’s odds of receiving neuraxial labor analgesia varied by state. Therefore, if a women moved to a state with a high probability of neuraxial labor analgesia, the odds of receiving neuraxial labor analgesia would increase by 50%. However, only a small percentage (ICC, 5.4%) of the total variance was explained by state. The MOR and ICC did not decrease after adjustment for patient-level factors (model 2). In model 3, the MOR was 1.4 (95% CI, 1.3-1.6), indicating that, after controlling for patient-level factors, anesthesia workforce measures explained only a small amount of state-level variation.

**Table 2.** Measures of Variation and Clustering in Neuraxial Labor Analgesia Use in the United States in 2015

Variable	Model 1^a^	Model 2^b^	Model 3^c^
State-level variance (SE)	0.173 (0.035)	0.186 (0.037)	0.147 (0.030)
MOR (95% CI)	1.5 (1.4-1.6)	1.5 (1.4-1.7)	1.4 (1.3-1.6)
ICC (95% CI), %	5.0 (3.5-7.4)	5.4 (4.0-7.9)	4.3 (2.8-6.4)

Abbreviations: ICC, intraclass correlation coefficient; MOR, median odds ratio.

^a^ Null model.

^b^ Adjusted for maternal age, race/ethnicity, insurance type, highest level of education, marital status, body mass index at delivery, prepregnancy diabetes, prepregnancy hypertension, prior live birth, previous cesarean delivery, trimester when prenatal care was initiated, gestational diabetes, gestational hypertension or preeclampsia, eclampsia, gestational age at delivery, labor augmentation, labor induction, birth attendant, and fetal presentation.

^c^ Adjusted for maternal age, race/ethnicity, insurance type, highest level of education, marital status, body mass index at delivery, prepregnancy diabetes, prepregnancy hypertension, prior live birth, previous cesarean delivery, trimester when prenatal care was initiated, gestational diabetes, gestational hypertension or preeclampsia, eclampsia, gestational age at delivery, labor augmentation, labor induction, birth attendant, number of anesthesiologists per 1000 births, and number of CRNAs per 1000 births.

### Subgroup Analysis

We performed subgroup analysis to examine the prevalence of neuraxial analgesia use according to mode of delivery. The unadjusted prevalences of neuraxial analgesia use among women who underwent vaginal delivery and intrapartum cesarean delivery were 71.1% and 89.7%, respectively. Heat maps and caterpillar plots of the prevalence of neuraxial analgesia use after adjusting for patient-level factors across US states among women who underwent vaginal delivery and intrapartum cesarean delivery are shown in eFigure 3 and eFigure 4 in the Supplement. Among the vaginal deliveries, the adjusted state-level prevalence ranged from 34.7% (95% CI, 31.4%-38.2%) in Maine to 78.6% (95% CI, 76.7%-80.3%) in Nevada. Among cesarean births, the adjusted state-level prevalence ranged from 26.9% (95% CI, 21.9%-32.9%) in South Dakota to 92.6% (95% CI, 91.5%-93.4%) in Idaho. The adjusted odds ratios derived from model 3 stratified according to delivery mode are listed in Table 3. In model 2 for vaginal delivery, we observed a MOR similar to that in our primary model (MOR, 1.5; 95% CI, 1.4-1.6). In model 2 for intrapartum cesarean delivery, we observed a greater degree of residual variation that was not explained by patient-level factors (MOR, 2.1; 95% CI, 1.8-2.4).

**Table 3.** Measures of Association Between Patient Characteristics, Anesthesia Workforce Measures, and Neuraxial Labor Analgesia Use Stratified by Delivery Mode in the United States in 2015

Variable	Adjusted OR (95% CI)	
	Vaginal Delivery (n = 2 344 368)^a^	Cesarean Delivery (n = 281 582)^a^
**Patient Characteristics**		
Maternal age, y		
<15	1.86 (1.66-2.08)	1.13 (0.73-1.75)
15-19	1.23 (1.22-1.25)	0.98 (0.92-1.03)
20-24	1.12 (1.11-1.13)	1.01 (0.97-1.05)
25-29	1 [Reference]	1 [Reference]
30-34	0.95 (0.94-0.95)	1.05 (1.02-1.09)
35-39	0.90 (0.89-0.91)	1.08 (1.03-1.13)
40-44	0.85 (0.83-0.86)	1.05 (0.97-1.14)
45-54	0.82 (0.75-0.90)	0.98 (0.76-1.25)
Race/ethnicity		
Non-Hispanic white	1 [Reference]	1 [Reference]
Hispanic	0.73 (0.73-0.74)	1.11 (1.06-1.15)
Non-Hispanic Asian	0.97 (0.96-0.99)	1.18 (1.11-1.25)
Non-Hispanic black	0.83 (0.82-0.84)	1.17 (1.12-1.21)
Non-Hispanic other	0.81 (0.79-0.82)	0.82 (0.77-0.88)
Insurance type		
Private	1 [Reference]	1 [Reference]
Medicaid	0.81 (0.80-0.81)	0.87 (0.84-0.90)
Other	0.94 (0.92-0.95)	0.95 (0.89-1.02)
Self-pay	0.54 (0.53-0.55)	0.72 (0.67-0.78)
Highest level of education		
Grade ≤8	0.56 (0.55-0.57)	0.69 (0.64-0.75)
Grade 9-12 with no diploma	0.87 (0.86-0.88)	0.95 (0.90-0.99)
High school graduate or GED completed	1 [Reference]	1 [Reference]
Associate degree	1.12 (1.10-1.13)	1.09 (1.03-1.14)
Some college credit but not a degree	1.14 (1.13-1.15)	1.07 (1.03-1.11)
Bachelor’s degree	1.14 (1.13-1.16)	1.20 (1.14-1.25)
Master’s degree	1.15 (1.13-1.16)	1.27 (1.20-1.35)
Doctorate or professional degree	1.23 (1.20-1.25)	1.27 (1.16-1.40)
Marital status		
Married	1 [Reference]	1 [Reference]
Unmarried	1.12 (1.11-1.13)	1.02 (0.99-1.05)
Body mass index at delivery^b^		
Normal or underweight	1 [Reference]	1 [Reference]
Overweight	1.09 (1.08-1.10)	1.08 (1.03-1.14)
Obesity class I	1.16 (1.14-1.17)	1.09 (1.03-1.15)
Obesity class II	1.22 (1.21-1.23)	1.06 (1.01-1.13)
Obesity class III	1.26 (1.25-1.28)	1.02 (0.96-1.08)
Prepregnancy diabetes	1.08 (1.03-1.13)	1.05 (0.94-1.16)
Prepregnancy hypertension	1.14 (1.10-1.17)	0.94 (0.87-1.02)
No prior live birth	1.62 (1.61-1.64)	1.42 (1.38-1.47)
Previous cesarean delivery	1.55 (1.52-1.58)	1.18 (1.12-1.23)
Trimester when prenatal care was initiated		
First	1 [Reference]	1 [Reference]
Second	0.87 (0.86-0.88)	0.90 (0.87-0.93)
Third	0.87 (0.86-0.88)	0.91 (0.85-0.96)
No prenatal care	0.60 (0.58-0.61)	0.90 (0.80-1.01)
Gestational diabetes	1.03 (1.01-1.04)	0.97 (0.92-1.01)
Gestational hypertension or preeclampsia	1.20 (1.18-1.22)	0.91 (0.88-0.95)
Eclampsia	1.15 (1.05-1.26)	0.89 (0.74-1.07)
Gestational age at delivery, wk		
37-42	1 [Reference]	1 [Reference]
24-36	0.82 (0.81-0.83)	0.74 (0.71-0.78)
<24	0.21 (0.20-0.23)	0.27 (0.17-0.41)
Labor augmentation	2.34 (2.32-2.35)	1.93 (1.87-2.00)
Labor induction	2.35 (2.33-2.37)	1.31 (1.27-1.35)
Birth attendant		
Physician	1 [Reference]	1 [Reference]
Midwife	0.58 (0.57-0.58)	0.74 (0.63-0.87)
Other	0.63 (0.61-0.65)	1.14 (0.80-1.64)
Fetal presentation		
Cephalic	1 [Reference]	1 [Reference]
Breech	0.69 (0.65-0.73)	0.77 (0.73-0.82)
Other	0.95 (0.92-0.97)	0.97 (0.90-1.04)
**Anesthesia Workforce Measures**		
No. of anesthesiologists per 1000 births^c^		
6.12-12.02	1 [Reference]	1 [Reference]
12.09-13.91	1.02 (0.71-1.46)	1.12 (0.55-2.32)
14.14-15.85	1.40 (0.98-1.99)	1.49 (0.77-2.88)
15.87-18.62	1.08 (0.75-1.56)	1.00 (0.52-1.92)
19.64-35.23	0.90 (0.63-1.28)	1.53 (0.79-2.98)
No. of CRNAs per 1000 births^c^		
4.58-8.49	1 [Reference]	1 [Reference]
8.53-12.41	0.89 (0.62-1.28)	0.70 (0.37-1.34)
12.62-21.19	1.10 (0.77-1.56)	0.99 (0.51-1.89)
23.32-29.87	0.93 (0.66-1.33)	0.88 (0.43-1.77)
30.19-38.31	1.02 (0.71-1.46)	0.64 (0.33-1.25)

Abbreviations: CRNAs, certified registered nurse anesthetists; GED, general equivalency diploma; OR, odds ratio.

^a^ Adjusted for maternal age, race/ethnicity, insurance type, highest level of education, marital status, body mass index at delivery, prepregnancy diabetes, prepregnancy hypertension, prior live birth, previous cesarean delivery, trimester when prenatal care was initiated, gestational diabetes, gestational hypertension or preeclampsia, eclampsia, gestational age at delivery, labor augmentation, labor induction, birth attendant, number of anesthesiologists per 1000 births, and number of CRNAs per 1000 births.

^b^ Body mass index (calculated as weight in kilograms divided by height in meters squared) categories are as follows: normal or underweight (<24.9), overweight (25-29.9), obesity class I (30-34.9), obesity class II (35-39.9), and obesity class III (≥40).

^c^ Ranges are quintiles.

## Discussion

In this study that included 2 625 950 women who underwent labor before giving birth, we identified substantial variation in the prevalence of neuraxial analgesia use, with a 2-fold difference in the highest prevalence state vs the lowest prevalence state. After adjusting for patient-level factors, only 5.4% of the statewide variation was attributable to the state. The MOR was greater than 1 in all models, suggesting that other unmeasured patient-level and hospital-level factors likely explain a large portion of the variance in neuraxial labor analgesia use across US states.

Our study could not determine why variation in neuraxial analgesia rates exists across states. One possible explanation for the residual variation is that the availability, type, and quality of obstetric anesthesia care in hospitals may vary among states. In states with a low prevalence of neuraxial analgesia use, such as Maine, Arkansas, Vermont, and Mississippi, a high proportion of the population lives in rural communities (61%, 44%, 61%, and 51%, respectively^21^). Research indicates that disparities exist in local access to obstetric services in vulnerable local communities^22^ and that rural hospitals increasingly face obstetric workforce challenges.^23^ Women who deliver in rural hospitals, which typically have low obstetric volumes,^24^ may face challenges in gaining access to obstetric anesthesia care. Based on data from the 2011 Obstetric Anesthesia Workforce Survey,^25^ hospitals with fewer than 500 annual births have lower rates of neuraxial analgesia compared with hospitals with 1500 or more annual births (66% vs 82%, respectively). In addition, only 15% of hospitals with fewer than 500 annual births have in-house obstetric anesthesia staff.^25^ In low-volume hospitals, especially those located in states with large rural populations, CRNAs more commonly deliver obstetric anesthesia care than physician anesthesiologists.^23,25^ There is a need for further research to determine whether limited access to obstetric anesthesia care, particularly in low-volume hospitals, explains the low prevalence of neuraxial analgesia use in rural US states.

Although we accounted for a number of patient-level factors previously shown to be associated with neuraxial analgesia use, such as race/ethnicity, insurance type, and highest level of education,^26,27,28,29,30,31^ other patient-level factors may account for some of the variation among states. For example, lower health literacy, cultural and religious beliefs, antenatal participation in childbirth education classes, and patient preferences may influence a patient’s decision to use neuraxial analgesia.^31,32^ Patients and obstetric care providers may not be aware of recent advances in neuraxial analgesia and the potential benefits of these advances on maternal outcomes.^33^ For example, neuraxial analgesia regimens using low concentrations of local anesthetic are associated with a lower risk of instrumental vaginal delivery compared with older regimens using higher-concentration solutions.^34,35^ Therefore, knowledge and biases about the effects of neuraxial analgesia among patients and health care professionals may vary by state. In addition, we could not assess whether states with high use rates of non–neuraxial analgesia techniques (eg, nitrous oxide) have low rates of neuraxial analgesia.

Findings from our secondary analysis of women who underwent vaginal delivery were consistent with our primary findings. However, we observed that women who underwent cesarean delivery had a high overall adjusted prevalence of neuraxial analgesia use (89.7%). Several reasons may explain this finding. Some women who received de novo neuraxial blockade for surgical anesthesia may have been misclassified as receiving neuraxial labor analgesia. Pregnant women in labor at risk of intrapartum cesarean delivery may have been advised to receive neuraxial analgesia to allow for later surgical anesthesia with an epidural top-up dose.^36^ Neuraxial labor analgesia is associated with cesarean delivery in observational studies but not in randomized clinical trials.^37^ A likely explanation is that the request for neuraxial analgesia is a marker for increased risk for cesarean delivery. Dysfunctional labor and a large and malpositioned fetus likely increase labor pain and the request for labor analgesia and are also associated with risk for cesarean delivery.^37^ For the subset of women herein with cesarean delivery, the range in the prevalence of neuraxial analgesia across states was wide (26.9%-92.6%), with a moderately high MOR of 2.1. The reason for these findings is uncertain; further work is needed to examine whether obstetric anesthetic practices differ across states for pregnant women in labor who are “at risk” for intrapartum cesarean delivery.

### Strengths and Limitations

The main strengths of our study are the large size of the data set comprising recent data on neuraxial analgesia. Evidence from the national data set also allowed us to account for a large number of patient-level factors in our multilevel model.

Nonetheless, the study has several limitations. First, despite concerns about the accuracy of diagnoses in birth certificates,^38,39,40^ prior evidence demonstrates high sensitivity (85%-96%) and a low false discovery rate (14%-20%) of neuraxial analgesia documentation in birth certificates.^38^ To our knowledge, specificity of neuraxial analgesia documentation has not been previously reported. However, we presume that the specificity is high because it is unlikely that neuraxial labor analgesia would be reported on a birth certificate for a patient who did not receive neuraxial labor analgesia. Second, despite the superior analgesia provided by neuraxial techniques, other potential direct and indirect effects of neuraxial analgesia on mother-centric outcomes (eg, satisfaction, postpartum recovery, and breastfeeding) have not been well studied. These outcomes are not captured in birth certificate data and thus could not be examined in our analysis. To model these outcomes, valid clinical data are required, as are other data for appropriate risk adjustment. Third, no studies to date have validated our statewide anesthesia workforce measures. These measures assume that all clinicians practice to a similar degree, despite differences in key attributes, such as level of training and years of experience. Another complicating factor is that CRNAs can practice independently in some states, but in other states physicians must supervise or direct CRNAs’ practice. Fourth, the study did not include hospital-level data; therefore, we could not determine the hospital type (teaching or community), frequency of neuraxial analgesia use at each hospital, or the number of anesthesia providers at the hospital level. Given that ACOG’s 2015 guidelines^3^ for the regionalization of maternal care state that anesthesia services should be available in all facilities, including those providing basic maternal care, further research is necessary to determine whether the prevalence of neuraxial analgesia use is a proxy for the availability of high-quality consultative obstetric anesthesia care.^41^ Fifth, we did not assess neuraxial labor analgesia use across US counties. An examination of county-level variation would provide finer resolution of the geographical variation of neuraxial labor analgesia use. Similarly, we did not have geographical data to determine the density of anesthesia providers across counties or other designated geographical zones.

## Conclusions

We examined national birth certificate data and found wide variation in the prevalence of neuraxial analgesia use across US states. Only a small portion of the overall variation was explained by state-level factors. Unmeasured patient-level and hospital-level factors likely account for a large portion of variance between states. With ACOG guidelines stating that anesthesia services should be available to provide labor analgesia in all hospitals that provide maternal care,^3,4^ efforts should be made to better understand the main reasons for the variation and whether this variation influences maternal or perinatal outcomes.

## Notes

Supplement. **eTable.** Frequencies of Missingness for Each Variable by State **eMethods.** Statistical Approach Used in the Random-Effects Models **eFigure 1.** Caterpillar Plot of Crude Prevalence of Neuraxial Analgesia by State **eFigure 2.** Caterpillar Plot of Adjusted Prevalence of Neuraxial Analgesia by State **eFigure 3.** Caterpillar Plots of Adjusted Prevalence of Neuraxial Analgesia by State for Vaginal Delivery and Cesarean Delivery Cohorts **eFigure 4.** Heat Maps of Adjusted Prevalence of Neuraxial Analgesia by State for Vaginal Delivery and Cesarean Delivery Cohorts
