# Supplementary material for: United States State-Level Variation in the Use of Neuraxial Analgesia During Labor for Pregnant Women
Source: JAMA Netw Open. 2018 Dec 28;1(8):e186567. doi: 10.1001/jamanetworkopen.2018.6567 (PMC6324365; doi:10.1001/jamanetworkopen.2018.6567)
Supplement: Supplement. — eTable. Frequencies of Missingness for Each Variable by State eMethods. Statistical Approach Used in the Random-Effects Models eFigure 1. Caterpillar Plot of Crude Prevalence of Neuraxial Analgesia by State eFigure 2. Caterpillar Plot of Adjusted Prevalence of Neuraxial Analgesia by State eFigure 3. Caterpillar Plots of Adjusted Prevalence of Neuraxial Analgesia by State for Vaginal Delivery and Cesarean Delivery Cohorts eFigure 4. Heat Maps of Adjusted Prevalence of Neuraxial Analgesia by State for Vaginal Delivery and Cesarean Delivery Cohorts [file jamanetwopen-1-e186567-s001.pdf]

Supplementary Online Content

Butwick AJ, Bentley J, Wong CA, Snowden JM, Sun E, Guo N. United States state-level variation in the use of neuraxial analgesia during labor for pregnant women. *JAMA Netw Open*. 2018;1(8):e186567. doi:10.1001/jamanetworkopen.2018.6567

eTable. Frequencies of Missingness for Each Variable by State

eMethods. Statistical Approach Used in the Random-Effects Models

eFigure 1. Caterpillar Plot of Crude Prevalence of Neuraxial Analgesia by State

eFigure 2. Caterpillar Plot of Adjusted Prevalence of Neuraxial Analgesia by State

eFigure 3. Caterpillar Plots of Adjusted Prevalence of Neuraxial Analgesia by State for Vaginal Delivery and Cesarean Delivery Cohorts

eFigure 4. Heat Maps of Adjusted Prevalence of Neuraxial Analgesia by State for Vaginal Delivery and Cesarean Delivery Cohorts

This supplementary material has been provided by the authors to give readers additional information about their work.

© 2018 Butwick AJ et al. *JAMA Network Open*.

eTable. Frequencies of Missingness for Each Variable by State

State	Race	Educn	Ins	BMI	GA	Prior live birth	Prev CD	PNC	PDM	PHTN	Ecl	GDM	GHTN	Fetal Presn	Birth Attdt	Total
**AK**	1.49	1.88	1.73	2.28	0.11	0.57	0.88	2.41	0.88	0.88	0.88	0.88	0.88	0.00	0.08	9.49
**AL**	0.00	0.26	0.00	2.05	0.03	0.00	0.00	0.06	0.00	0.00	0.00	0.00	0.00	0.05	0.00	2.40
**AR**	0.26	0.68	3.87	3.52	0.05	0.11	0.00	15.92	0.00	0.00	0.00	0.00	0.00	0.00	0.07	21.36
**AZ**	0.51	0.43	0.29	0.30	0.10	0.05	0.00	1.90	0.00	0.00	0.00	0.00	0.00	0.10	0.02	3.14
**CA**	2.09	4.42	0.10	2.33	0.10	0.09	0.00	1.56	0.00	0.00	0.00	0.00	0.00	1.46	0.01	8.94
**CO**	1.24	0.91	0.10	2.07	0.02	0.05	0.00	2.11	0.00	0.00	0.00	0.00	0.00	0.00	0.10	5.76
**DC**	0.23	1.18	0.85	2.56	0.03	0.01	0.07	2.13	0.07	0.07	0.07	0.07	0.07	0.00	0.00	5.70
**DE**	0.04	0.58	0.21	2.34	0.14	0.03	0.00	1.16	0.00	0.00	0.00	0.00	0.00	0.00	0.13	3.81
**FL**	0.24	0.67	0.26	4.51	0.03	0.63	0.13	4.77	0.13	0.13	0.13	0.13	0.13	0.03	0.00	9.62
**GA**	1.31	1.47	0.31	7.01	0.04	5.60	0.42	14.00	0.42	0.42	0.42	0.42	0.42	0.42	0.01	19.94
**HI**	0.10	0.55	0.65	1.93	0.05	0.01	0.00	3.93	0.00	0.00	0.00	0.00	0.00	3.62	0.26	9.53
**IA**	0.00	0.11	0.04	0.30	0.02	0.02	0.00	0.25	0.00	0.00	0.00	0.00	0.00	0.00	0.00	0.71
**ID**	0.27	0.40	0.09	0.29	0.01	0.10	0.03	0.35	0.03	0.03	0.03	0.03	0.03	0.10	0.00	1.42
**IL**	0.92	1.59	0.28	1.34	0.05	0.34	0.09	3.97	0.09	0.09	0.09	0.09	0.09	0.15	0.03	7.42
**IN**	0.04	0.11	0.10	0.19	0.02	0.02	0.00	0.13	0.00	0.00	0.00	0.00	0.00	0.14	0.03	0.74
**KS**	0.09	0.23	0.47	0.12	0.04	0.00	0.00	0.05	0.00	0.00	0.00	0.00	0.00	0.00	0.00	0.91
**KY**	0.12	0.32	0.55	1.10	0.02	0.01	0.27	3.47	0.27	0.27	0.27	0.27	0.27	0.07	0.00	4.98
**LA**	0.11	0.36	0.00	1.74	0.04	2.68	0.00	2.57	0.00	0.00	0.00	0.00	0.00	0.00	0.03	6.89

© 2018 Butwick AJ et al. *JAMA Network Open*.

**MA**	1.59	2.42	6.22	3.67	0.02	0.05	0.00	1.99	0.00	0.00	0.00	0.00	0.00	4.29	0.02	16.65
**MD**	0.31	0.53	0.77	2.95	0.07	1.54	0.00	7.02	0.00	0.00	0.00	0.00	0.00	0.63	0.11	12.61
**ME**	0.00	0.12	0.42	0.12	0.04	0.18	0.00	0.13	0.00	0.00	0.00	0.00	0.00	0.27	0.00	1.19
**MI**	0.16	0.79	0.15	1.24	0.03	0.06	0.27	2.57	0.27	0.27	0.27	0.27	0.27	0.53	0.00	5.05
**MN**	0.61	1.16	0.11	1.40	0.05	0.06	0.12	0.96	0.12	0.12	0.12	0.12	0.12	0.06	0.19	3.68
**MO**	0.37	0.25	1.03	2.80	0.10	0.35	0.00	4.25	0.00	0.00	0.00	0.00	0.00	0.40	0.00	8.29
**MS**	0.01	0.13	0.05	0.28	0.02	0.00	0.02	1.58	0.02	0.02	0.02	0.02	0.02	0.02	0.00	1.95
**MT**	0.27	0.37	0.27	0.46	0.03	0.00	0.02	0.36	0.02	0.02	0.02	0.02	0.02	0.00	0.00	1.61
**NC**	0.04	0.23	0.00	1.61	0.04	0.01	0.00	0.81	0.00	0.00	0.00	0.00	0.00	0.00	0.20	2.78
**ND**	1.24	1.25	3.46	0.49	0.01	0.14	0.00	3.59	0.00	0.00	0.00	0.00	0.00	0.00	0.00	9.07
**NE**	0.03	0.07	1.46	1.22	0.06	0.12	0.20	2.32	0.20	0.20	0.20	0.20	0.20	0.00	0.01	5.13
**NH**	0.67	0.67	1.17	4.22	0.13	0.68	1.15	0.23	1.15	1.15	1.15	1.15	1.15	0.00	0.00	7.97
**NJ**	1.55	0.56	0.00	0.95	0.04	0.17	0.00	3.12	0.00	0.00	0.00	0.00	0.00	0.00	0.01	6.08
**NM**	1.22	1.33	6.11	3.06	0.04	1.07	0.00	4.22	0.00	0.00	0.00	0.00	0.00	0.08	0.00	14.71
**NV**	0.25	1.74	0.88	1.54	0.01	0.23	0.00	2.82	0.00	0.00	0.00	0.00	0.00	0.00	0.00	6.42
**NY**	1.60	0.40	0.12	1.10	0.00	0.22	0.05	1.88	0.05	0.05	0.05	0.05	0.05	0.05	0.00	4.94
**OH**	0.42	0.70	0.62	2.43	0.06	0.49	0.00	1.73	0.00	0.00	0.00	0.00	0.00	0.08	0.00	5.61
**OK**	0.14	0.31	0.31	1.19	0.07	0.18	0.00	2.18	0.00	0.00	0.00	0.00	0.00	0.00	0.00	3.63
**OR**	0.43	0.39	0.14	0.46	0.04	0.13	0.00	0.57	0.00	0.00	0.00	0.00	0.00	0.06	0.00	1.99
**PA**	1.18	0.62	2.64	9.87	0.15	0.92	0.00	3.97	0.00	0.00	0.00	0.00	0.00	0.00	0.00	17.00
**RI**	0.79	10.80	0.10	5.00	0.02	1.56	0.08	10.71	0.08	0.08	0.08	0.08	0.08	0.03	0.05	23.62
**SC**	0.23	0.30	0.52	0.95	0.05	0.04	0.00	0.13	0.00	0.00	0.00	0.00	0.00	0.00	0.05	2.05

© 2018 Butwick AJ et al. *JAMA Network Open*.

**SD**	0.14	0.11	0.22	0.90	0.03	0.02	0.18	1.37	0.18	0.18	0.18	0.18	0.18	0.04	0.00	2.66
**TN**	0.15	0.50	2.38	5.18	0.17	0.65	0.00	8.05	0.00	0.00	0.00	0.00	0.00	0.00	0.00	14.15
**TX**	0.08	0.13	0.15	0.38	0.03	0.05	0.00	2.27	0.00	0.00	0.00	0.00	0.00	0.87	0.05	3.79
**UT**	3.04	2.57	5.22	0.84	0.00	0.05	0.00	1.73	0.00	0.00	0.00	0.00	0.00	0.00	0.00	10.91
**VA**	0.12	2.32	0.01	12.32	0.03	0.02	0.00	14.04	0.00	0.00	0.00	0.00	0.00	36.61	0.01	47.11
**VT**	1.53	0.69	0.53	1.35	0.07	0.09	0.00	0.64	0.00	0.00	0.00	0.00	0.00	0.75	0.00	4.79
**WA**	1.64	1.03	1.55	3.42	0.06	0.79	0.08	5.05	0.08	0.08	0.08	0.08	0.08	0.23	0.05	10.80
**WI**	0.37	0.35	0.52	1.68	0.23	0.15	0.18	3.10	0.18	0.18	0.18	0.18	0.18	0.19	0.00	5.73
**WV**	0.61	0.37	0.25	1.96	0.06	1.71	0.41	1.04	0.41	0.41	0.41	0.41	0.41	0.25	0.02	6.09
**WY**	1.73	0.85	0.56	2.20	0.00	0.02	0.00	1.94	0.00	0.00	0.00	0.00	0.00	0.00	0.02	6.08
**All**	0.78	1.18	0.69	2.54	0.06	0.48	0.06	3.33	0.06	0.06	0.06	0.06	0.06	1.34	0.03	8.43

Attdt = Attendant; BMI = body mass index; CD = cesarean delivery; Ecl = eclampsia; GDM = gestational diabetes; GHTN = gestational hypertension; GA = gestational age at delivery; Ins = insurance; PDM = prepregnancy diabetes; PHTN = prepregnancy hypertension; PNC = prenatal care; Presn = presentation

© 2018 Butwick AJ et al. *JAMA Network Open*.

eMethods. Statistical Approach Used in the Random-Effects Models

Multilevel logistic regression was fit by the GLMIMIX procedure in SAS 9.4 (SAS Institute Inc, Cary, NC) using maximum likelihood estimation based on Laplace approximation. Three regression models were fit sequentially. First, we developed an unconditional or 'null' model including the state identifier as a random effect. In the null model, the random effect may be interpreted as each state's deviation from the mean state-level neuraxial analgesia rate. In the second model, patient-level factors were added to determine how much of the variation can be explained by patient-level factors. In the third model, state-level anesthesiologists and certified registered nurse anesthetists (CRNA) workforce measures were added, whilst controlling for individual-level determinants of neuraxial analgesia use. Because of a non-linear relationship between each state-level workforce measure and our outcome, we categorized each workforce measure by quintiles; this categorization also allows for greater ease of data interpretation. The adjusted state-specific prevalence of neuraxial analgesia can be interpreted as each state's deviation from the mean neuraxial analgesia rate that is not accounted for by covariates. For each model, the state-level neuraxial analgesia prevalence and 95% confidence interval (CI) were calculated by first converting each state's estimated odds ratio for neuraxial labor analgesia obtained from the multilevel logistic regression model (exponential of random effect) into a relative risk and then multiplying by the national rate.[1] The association between individual patient-level variables and neuraxial analgesia use was measured by the regression coefficients and 95% CI.

In order to quantify and interpret the variance across states, we calculated the median odds ratio (MOR). The MOR estimates the odds of neuraxial analgesia that would occur if a patient moved from a state with a low odds of neuraxial analgesia to a state with a high odds of

© 2018 Butwick AJ et al. *JAMA Network Open*.

neuraxial analgesia, after case-mix adjustment.[2] The MOR is computed by translating the state-level variance into an odds scale. $MOR = \exp[\sqrt{2 \times V_A} \times 0.6745]$ (V_A = area residual variance on the logistic scale).[2] In addition, we compared the MORs across models (null model vs. adjusted model) to examine the extent to which variables explain variance in the odds of the intervention across states. If the MOR=1, then no variation exists among states; if the MOR>1, variation is present among states, with the variation increasing as the MOR increases. We calculated the intraclass correlation coefficient (ICC) to estimate the proportion of total variance in neuraxial labor analgesia that is attributable to the state level using the latent variable method: $ICC = V_A/(V_A + 3.29)$.[2] To obtain 95% confidence intervals for the MOR and ICC we applied the formulas for each to the upper and lower 95% confidence limits of the random-effects variance estimated by profile-likelihood.[3,4]

References

1. Larsen K, Petersen JH, Budtz-Jorgensen E, Endahl L. Interpreting parameters in the logistic regression model with random effects. Biometrics 2000;56:909-14.
2. Merlo J, Chaix B, Ohlsson H, Beckman A, Johnell K, Hjerpe P, Rastam L, Larsen K. A brief conceptual tutorial of multilevel analysis in social epidemiology: using measures of clustering in multilevel logistic regression to investigate contextual phenomena. J Epidemiol Community Health 2006;60:290-7.
3. Saha KK. Profile likelihood-based confidence interval of the intraclass correlation for binary outcome data sampled from clusters. Stat Med 2012; 31: 3982-4002.
4. Zou G, Donner A. Confidence interval estimation of the intraclass correlation coefficient for binary outcome data. Biometrics 2004; 60: 807-11.

© 2018 Butwick AJ et al. *JAMA Network Open*.

eFigure 1. Caterpillar Plot of Crude Prevalence of Neuraxial Analgesia by State

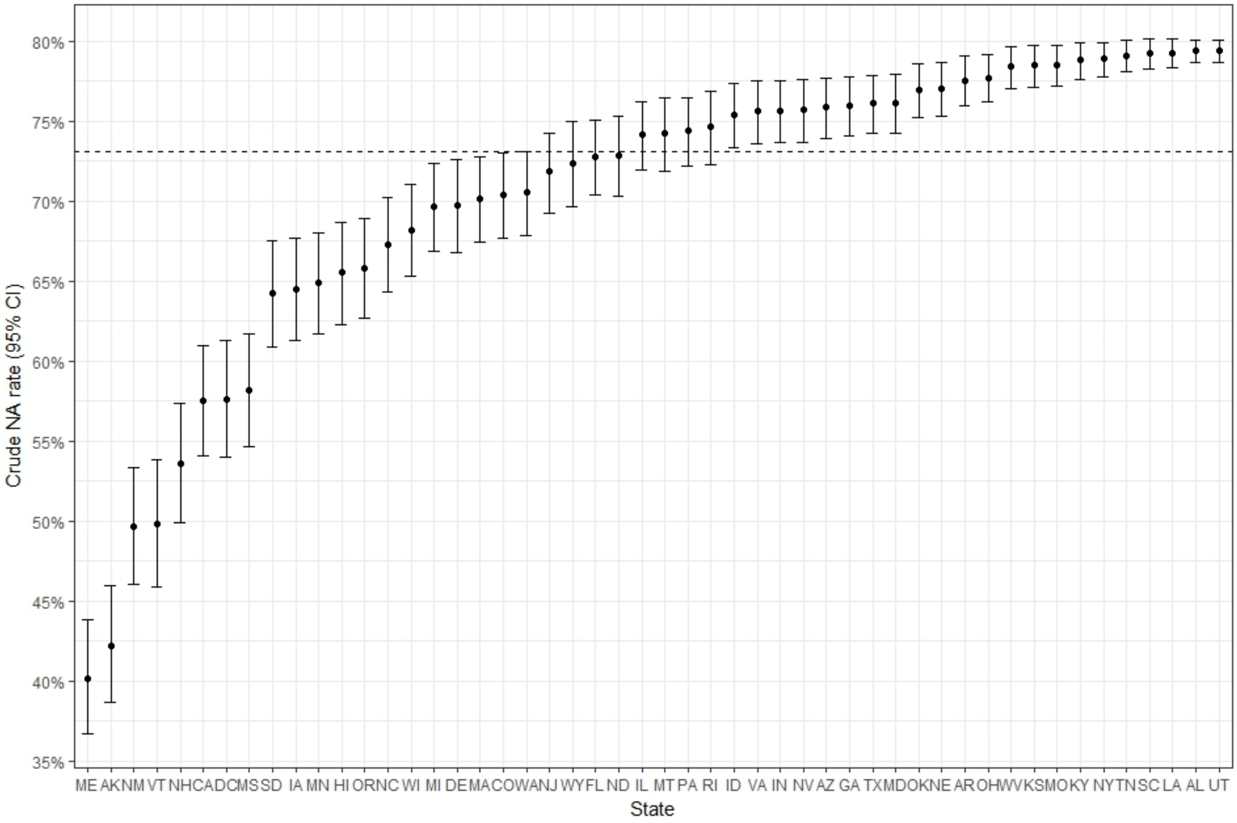

CI = confidence intervals; NA = neuraxial analgesia
Dotted horizontal line indicates the mean crude prevalence of neuraxial analgesia among all states

© 2018 Butwick AJ et al. *JAMA Network Open*.

eFigure 2. Caterpillar Plot of Adjusted Prevalence of Neuraxial Analgesia by State

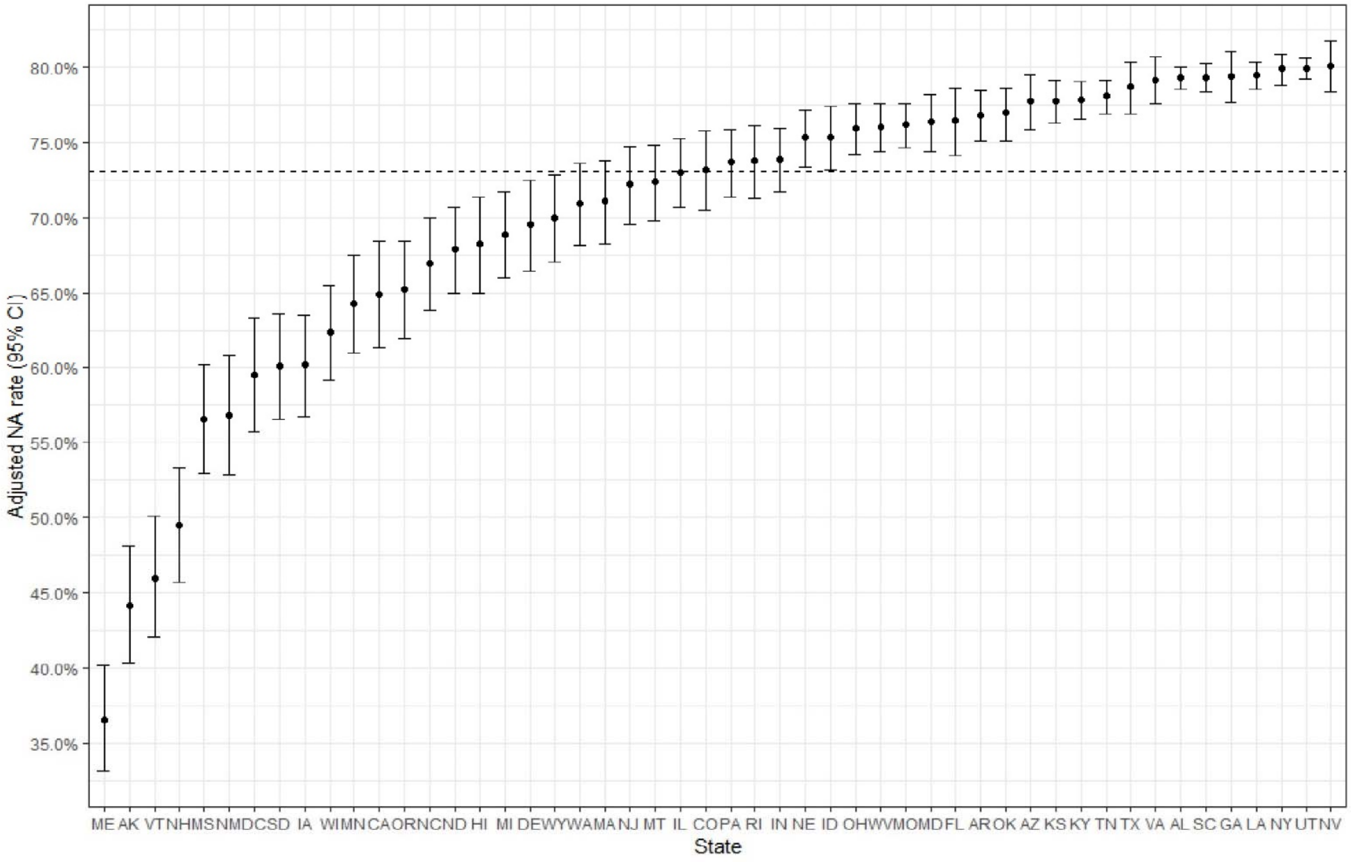

CI = confidence intervals; NA = neuraxial analgesia
Dotted horizontal line indicates the mean adjusted prevalence of neuraxial analgesia among all states

© 2018 Butwick AJ et al. *JAMA Network Open*.

eFigure 3. Caterpillar Plots of Adjusted Prevalence of Neuraxial Analgesia by State for Vaginal Delivery and Cesarean Delivery Cohorts

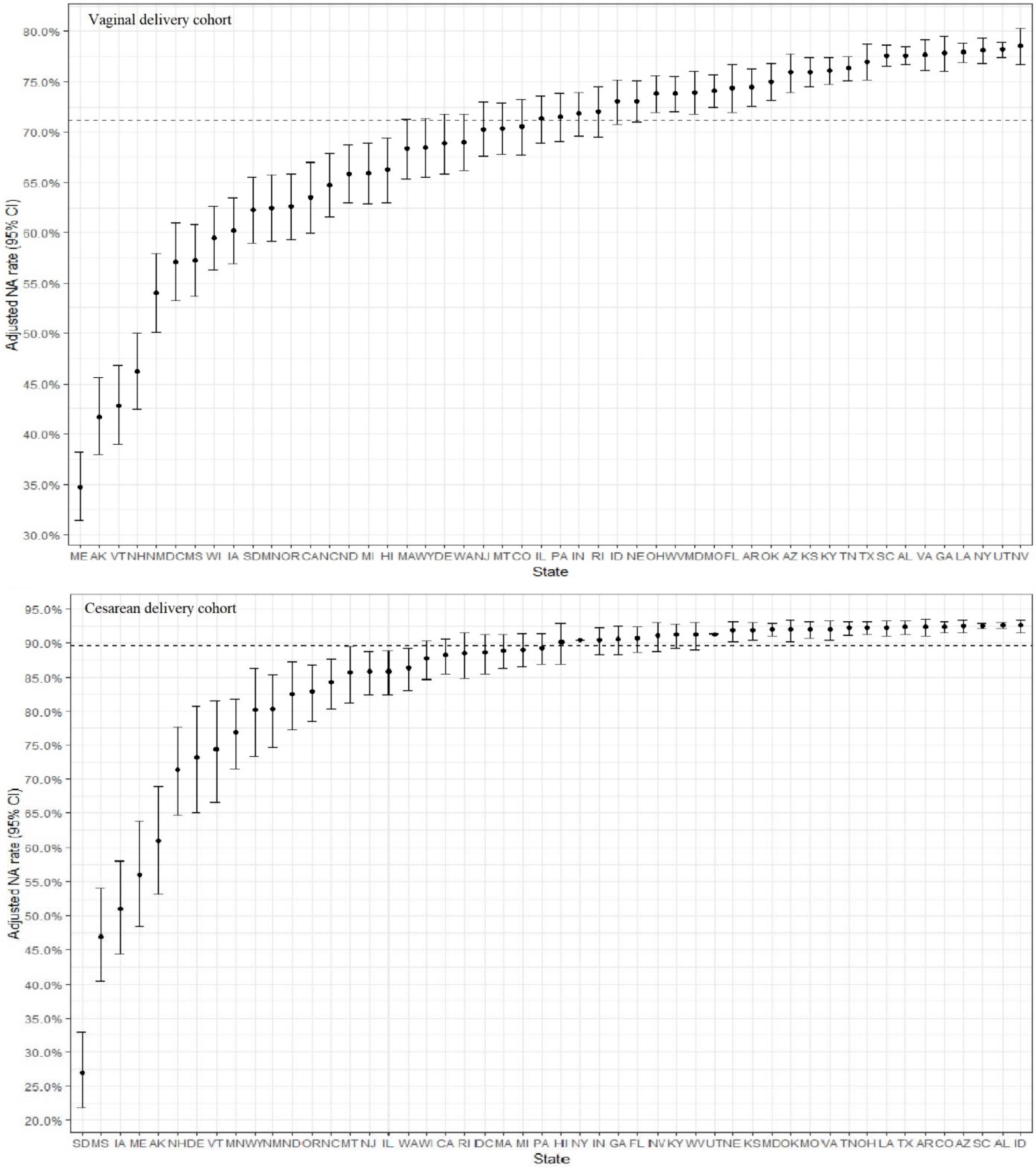

CI = confidence intervals; NA = neuraxial analgesia
Dotted horizontal line indicates the mean adjusted prevalence of neuraxial analgesia among all states

© 2018 Butwick AJ et al. *JAMA Network Open*.

eFigure 4. Heat Map of Adjusted Prevalence of Neuraxial Analgesia by State for Vaginal Delivery and Cesarean Delivery Cohorts

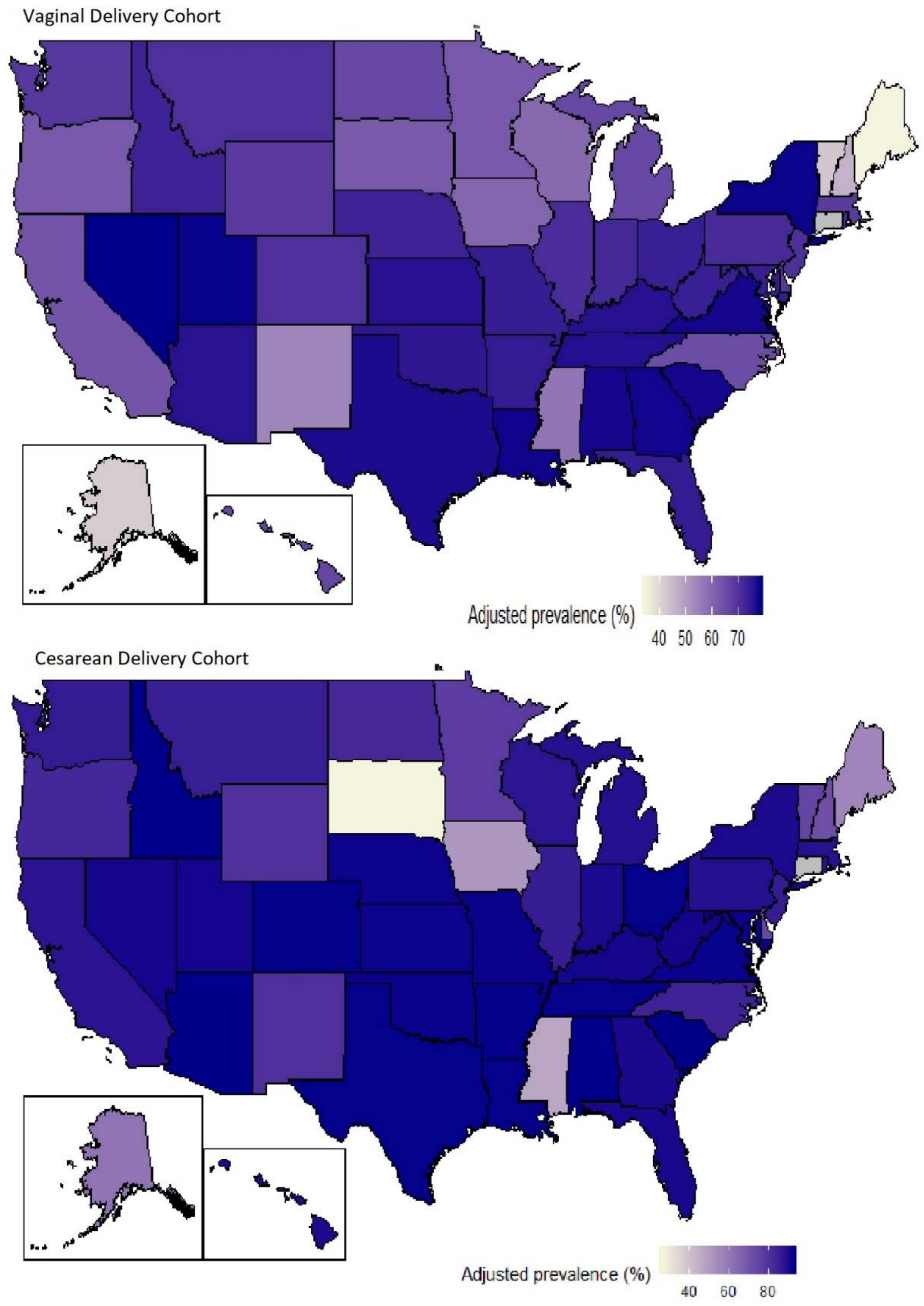

Connecticut did not provide birth data for 2015 and is depicted in grey on the heat maps.

© 2018 Butwick AJ et al. *JAMA Network Open*.
